# A dinuclear silver(I) com­plex of 6-phenyl-2,2′:6′,2′′-terpyridine

**DOI:** 10.1107/S2414314626010436

**Published:** 2026-10-15

**Authors:** Yuto Nakaie, Tomoya Abe, Yosuke Hosoya, Kosuke Sugawa, Joe Otsuki

**Affiliations:** a Department of Applied and Materials Chemistry College of Science and Technology Nihon University, 1-8-14 Kanda Surugadai Chiyoda-ku Tokyo 101-8308 Japan; Katholieke Universiteit Leuven, Belgium

**Keywords:** argentophilic inter­action, crystal structure, dinuclear com­plex, π–π stacking, silver(I), terpyridine

## Abstract

A dinuclear silver(I) com­plex of 6-phenyl-2,2′:6′,2′′-terpyridine, bis­(μ-6-phenyl-2,2′:6′,2′′-terpyridine)­bis­[(tri­fluoro­methane­sulfonato)­silver(I)], [Ag_2_(C_21_H_15_N_3_)(CF_3_SO_3_)_2_], was obtained by crystallization with silver tri­fluoro­methane­sulfonate and characterized by single-crystal X-ray diffraction. Two symmetry-related terpyridine ligands bridge two Ag^I^ ions. The Ag^I^ ion has a highly distorted four-coordinate N_3_O environment com­prising three pyridyl N atoms and one tri­fluoro­methane­sulfonate O atom. The intra­molecular Ag⋯Ag separation of 2.8501 (8) Å is consistent with an argentophilic inter­action. An intra­molecular phen­yl–pyridyl π–π contact is also present. The structure demonstrates that a single phenyl substituent at the 6-position is com­patible with ligand bridging and the formation of a short Ag⋯Ag contact.

## Abstract 2

A dinuclear silver(I) com­plex of 6-phenyl-2,2′:6′,2′′-terpyridine features bridging terpyridine ligands and a short intra­molecular Ag⋯Ag contact indicative of an argentophilic inter­action.

## Graphical abstract

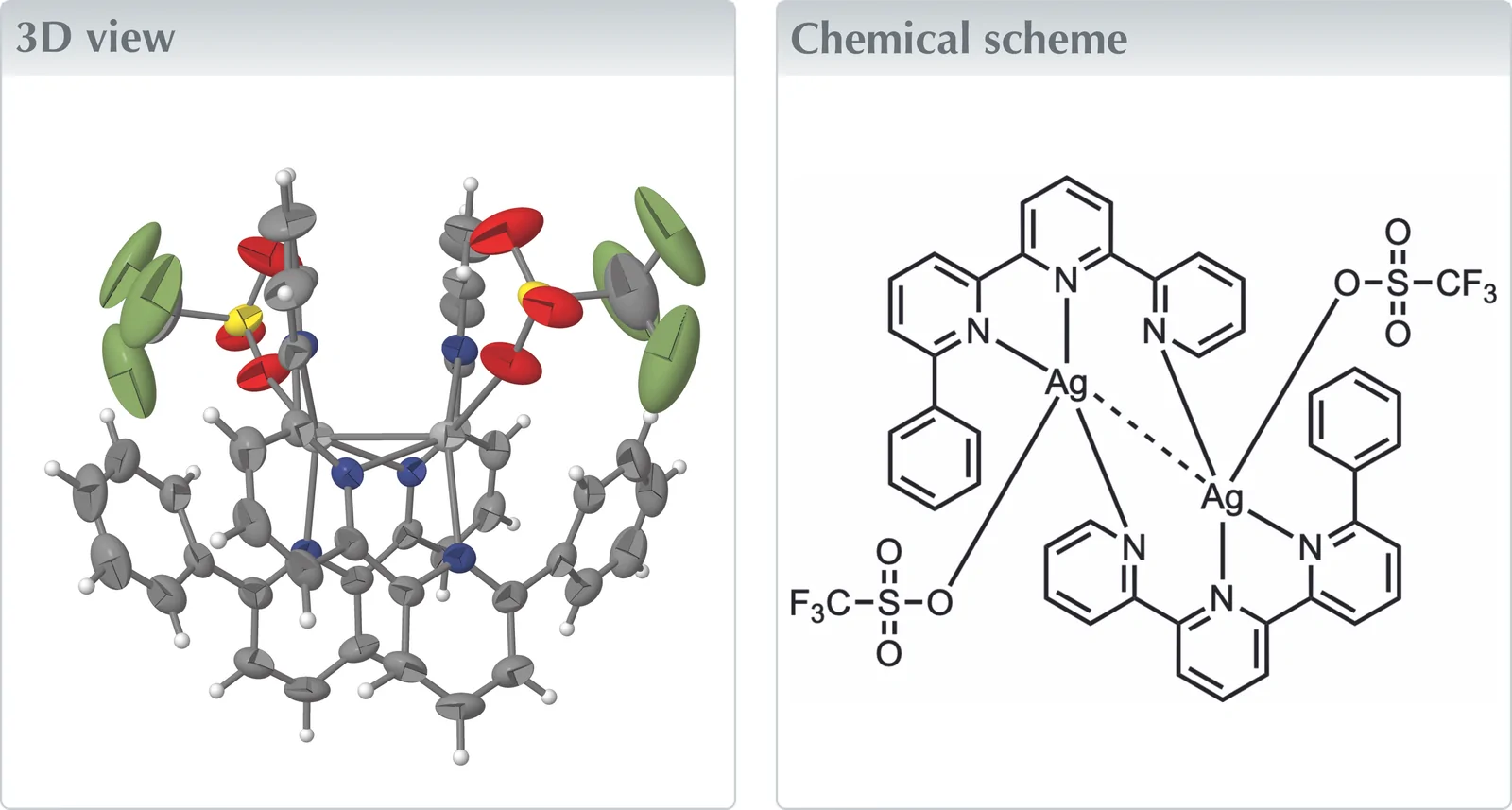

## Structure description

Ag^I^ com­plexes of 2,2′:6′,2′′-terpyridine (tpy) exhibit diverse coordination modes and solvent-dependent nuclearity. Crystallization from aceto­nitrile (MeCN) affords approximately square-planar [Ag(tpy)(MeCN)]^+^ units, which associate into dimers through an Ag⋯Ag contact of 3.170 Å (Constable *et al.*, 1998*a* ▸,*b* ▸). Chiral tpy derivatives form mononuclear aceto­nitrile com­plexes but favour dinuclear double helicates in less coordinating solvents. These helicates contain intra­molecular Ag⋯Ag contacts of 2.914–2.940 Å and associate into tetra­nuclear aggregates through additional Ag⋯Ag inter­actions (Baum *et al.*, 1998 ▸). Similarly, 6,6′′-dimethyl-tpy forms dinuclear double helicates with Ag⋯Ag distances of 2.930–2.970 Å. Their structures depend on the counter-ion: nitrate and tri­fluoro­methane­sulfonate produce asymmetric helicates with one bridging central pyridine N atom, whereas BF_4_^−^ and ClO_4_^−^ give more symmetrical structures with both central N atoms bridging the Ag pair (Fik *et al.*, 2014 ▸). Dinuclear Ag–tpy units can also form extended chains with gold com­plexes through Ag⋯Au and Au⋯Au inter­actions (Gil-Moles *et al.*, 2017 ▸). Solution studies support mononuclear [Ag(tpy)_2_]^+^ species. Thermodynamic measurements in dimethyl sulfoxide (DMSO) suggest five-coordinate bonds with one unbound pyridine ring (Del Piero *et al.*, 2007 ▸), while selective formation of an [Ag(tpy)_2_]^+^ unit was demonstrated in an Ag^I^ capture–release system (Paul *et al.*, 2019 ▸).

**Figure 1:**
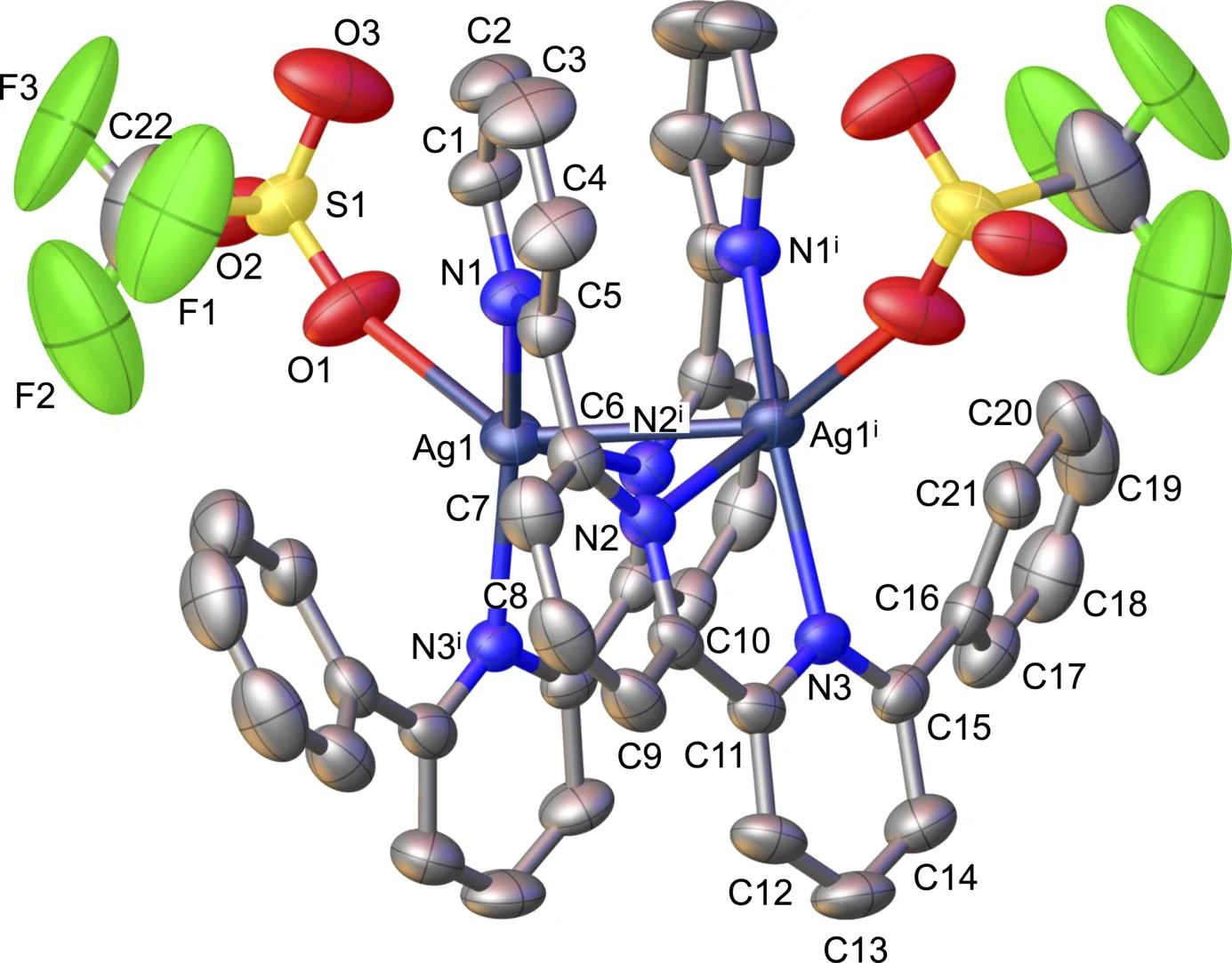
The mol­ecular structure of the dinuclear Ag^I^ com­plex of 6-Phtpy. Displacement ellipsoids are drawn at the 50% probability level. H atoms have been omitted for clarity. [Symmetry code: (i) −*x* + 1, *y*, −*z* + 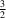.]

Against this background, we report the crystal structure of the tri­fluoro­methane­sulfonate salt of a dinuclear Ag^I^ com­plex containing 6-phenyl-2,2′:6′,2′′-terpyridine (6-Phtpy). The asymmetric unit contains one 6-Phtpy ligand, one Ag^I^ ion and one tri­fluoro­methane­sulfonate anion. Two symmetry-related 6-Phtpy ligands bridge two central Ag^I^ ions in a helical arrangement, forming a chiral dinuclear com­plex (Fig. 1 ▸). A crystallographic twofold rotation axis passes through the midpoint of, and perpendicular to, the Ag⋯Ag vector. Each Ag^I^ centre has a highly distorted four-coordinate N_3_O environment containing one pyridyl N atom from one ligand, two pyridyl N atoms from the other ligand and one tri­fluoro­methane­sulfonate O atom. The Ag1—N1, Ag1—N2^i^ and Ag1—N3^i^ distances are 2.240 (3), 2.412 (3) and 2.330 (2) Å, respectively, and Ag1—O1 is 2.578 (4) Å [symmetry code: (i) −*x* + 1, *y*, −*z* + 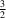]. The intra­molecular Ag1⋯Ag1^i^ distance of 2.8500 (6) Å is consistent with an argentophilic inter­action (Schmidbaur & Schier, 2015 ▸). The ligand adopts a nonplanar conformation. The dihedral angles between the N1- and N2-containing pyridine rings and between the N2- and N3-containing rings are 43.85 (16) and 16.62 (13)°, respectively. The phenyl substituent is inclined by 38.65 (16)° to the adjacent N3-containing pyridine ring. Within the dinuclear com­plex, the phenyl ring and a symmetry-related N2^i^-containing pyridine ring form a π–π contact, with a centroid–centroid distance of 3.750 (3) Å and an inter­planar angle of 10.58 (15)°.

The dinuclear framework resembles that of Ag_2_(tpy)_2_, which contains bridging unsubstituted tpy ligands and weak Ag—O inter­actions with tri­fluoro­methane­sulfonate anions (Gil-Moles *et al.*, 2017 ▸). The reported Ag_2_(tpy)_2_ com­plex has an Ag⋯Ag contact of 3.1698 (12) Å. The related [Ag(dptpy)(MeCN)]^+^ com­plex, with dptpy = 6,6′′-diphenyl-tpy, is mononuclear and has no short Ag⋯Ag contact; the shortest separation is 5.656 Å (Constable *et al.*, 1998 ▸). Thus, a single phenyl substituent at the 6-position is com­patible with ligand bridging and a short intra­molecular Ag⋯Ag contact.

**Figure 2:**
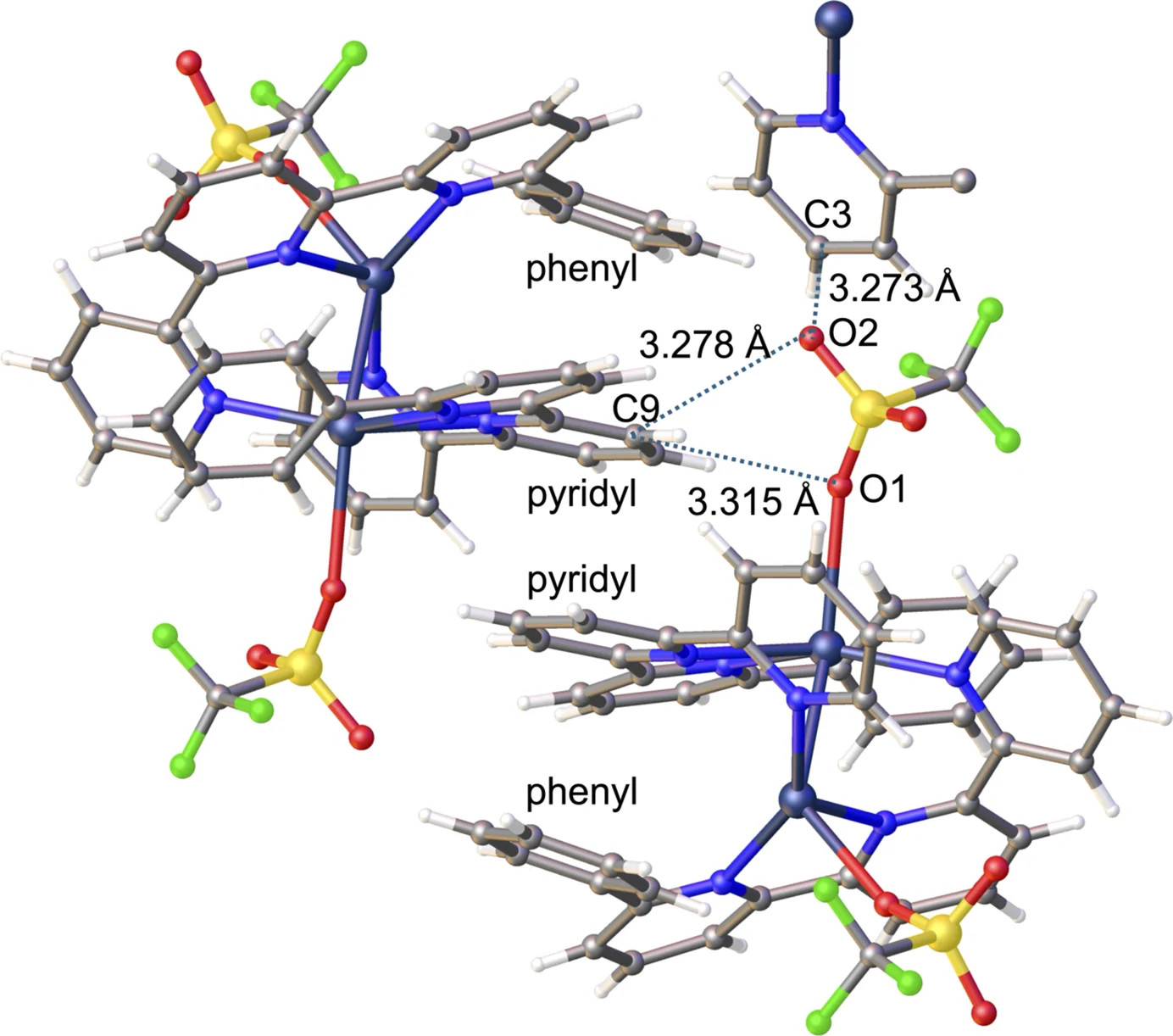
The crystal packing of the dinuclear com­plex, showing phen­yl–pyrid­yl–pyrid­yl–phenyl π–π stacking and C—H⋯O contacts (labelled distances are C⋯O separations).

The crystal packing features offset π–π stacking between the central pyridine rings of neighbouring com­plexes, together with weak C—H⋯O contacts involving the tri­fluoro­methane­sulfonate anions (Fig. 2 ▸). The central pyridine rings of neighbouring com­plexes of opposite handedness, related by inversion (symmetry operation: −*x*, −*y* + 1, −*z* + 2), are parallel, with a centroid–centroid distance of 3.879 (3) Å. The C3—H3⋯O2 contact has a C⋯O distance of 3.273 (6) Å and a C—H⋯O angle of 150°. The H9 atom forms a bifurcated contact with O2 and O1, with C9⋯O distances of 3.278 (5) and 3.315 (5) Å, and angles of 132 and 130°, respectively. Details of the weak C—H⋯O contacts are given in Table 1 ▸.

## Synthesis and crystallization

**Figure 3:**
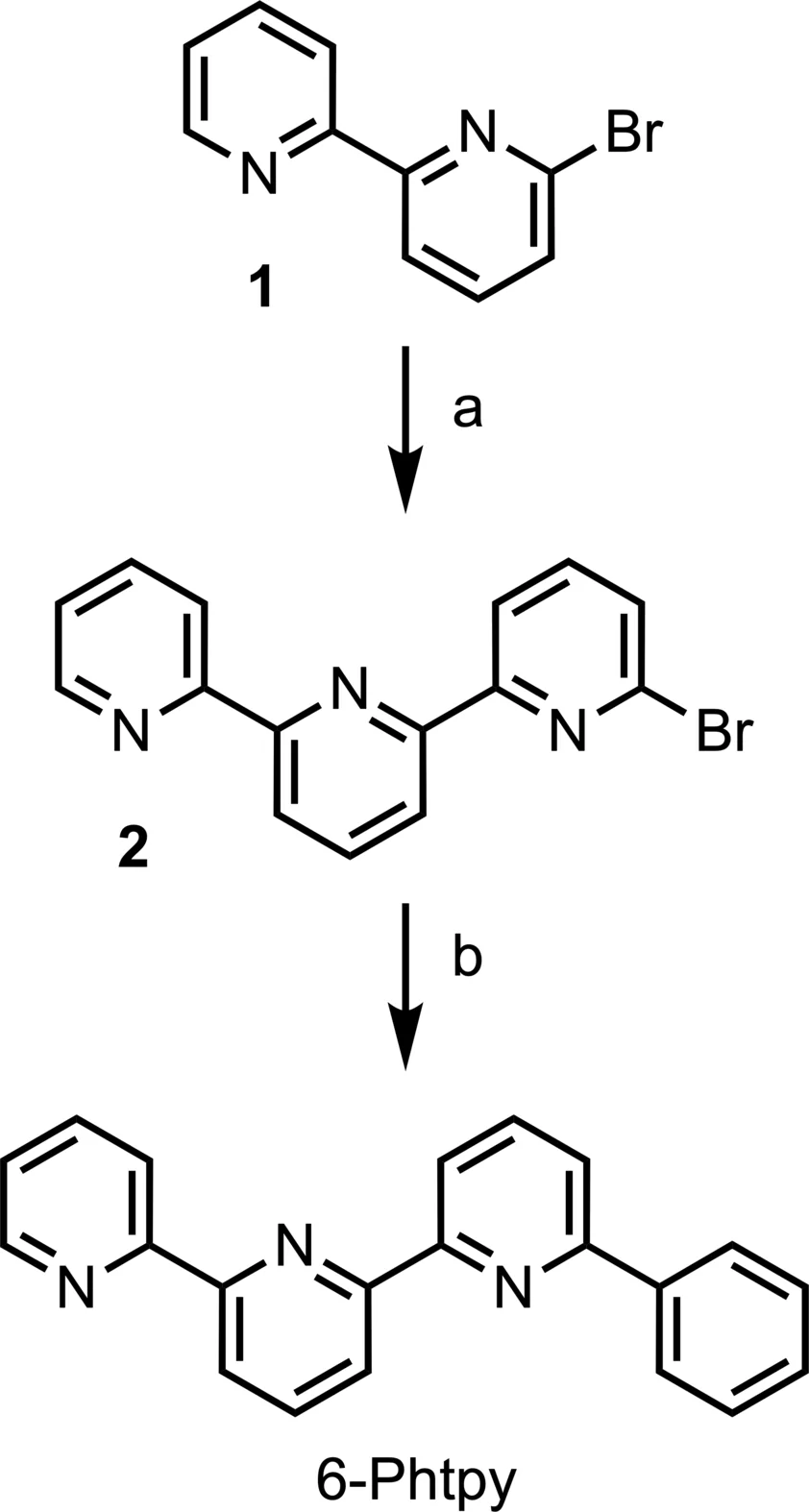
Synthetic route to 6-Phtpy. Reagents and conditions: (*a*) i) hexa­methyl­ditin, Pd(PPh_3_)_4_, DME, reflux, 18 h; ii) 2,6-di­bromo­pyridine, Pd(PPh_3_)_4_, toluene, reflux, 24 h, 45%; (*b*) phenyl­boronic acid pinacol ester, Pd(PPh_3_)_4_, Na_2_CO_3_, H_2_O, toluene, EtOH, reflux, 24 h, 85%.

The synthetic route to 6-Phtpy is shown in Fig. 3 ▸.

**6-Bromo-2,2′:6′,2′′-terpyridine (2).** The known com­pound **2** was prepared according to literature procedures (Derrick *et al.*, 2020 ▸; Stefankiewicz *et al.*, 2013 ▸) using solvents without prior drying and with the purification procedure modified as follows. The product was purified by column chromatography (SiO_2_, CHCl_3_), followed by gel permeation chromatography (GPC), to afford com­pound **2** in 45% yield.

**6-Phenyl-2,2′:6′,2′′-terpyridine (6-Phtpy).** 6-Phtpy is a known com­pound (Constable *et al.*, 1999 ▸), but its synthesis from com­pound **2** has not been reported previously. Compound **2** (168 mg, 0.537 mmol), phenyl­boronic acid pinacol ester (115 mg, 0.563 mmol), Pd(PPh_3_)_4_ (65 mg, 0.056 mmol) and Na_2_CO_3_ (286 mg, 2.69 mmol) were added to a mixture of H_2_O (1.3 ml), toluene (5.0 ml) and EtOH (2.5 ml). The mixture was degassed by three freeze–pump–thaw cycles and refluxed under N_2_ for 24 h. The solvents were removed under reduced pressure, and the residue was dissolved in CH_2_Cl_2_. The solution was washed with water three times, and the organic layer was concentrated under reduced pressure. The crude product was washed with MeOH and collected by filtration. The resulting solid was dissolved in CHCl_3_, and the solution was filtered. The filtrate was concentrated under reduced pressure to afford 6-Phtpy as a white solid (yield: 142 mg, 0.458 mmol, 85%).

**Crystallization.** 6-Phtpy and silver(I) tri­fluoro­methane­sulfonate (AgOTf) were dissolved in acetone (10 ml) in a 10 ml vial, giving a concentration of 2.5 m*M* for each com­ponent. Slow evaporation at room tem­per­a­ture under ambient conditions afforded colourless needle-shaped crystals after 44 d. A block-shaped specimen was cut from a needle for single-crystal X-ray diffraction.

## Refinement

Crystal data, data collection and structure refinement details are summarized in Table 2 ▸. Reflections 200 and 110 were omitted because they were considered to be affected by the beamstop. The tri­fluoro­methane­sulfonate CF_3_ group exhibits large anisotropic displacement parameters. Inspection of the difference electron-density map revealed no significant residual features near this group.

## Supplementary Material

Crystal structure: contains datablock(s) I. DOI: 10.1107/S2414314626010436/vm4086sup1.cif

Structure factors: contains datablock(s) I. DOI: 10.1107/S2414314626010436/vm4086Isup2.hkl

CCDC reference: 2594042

Additional supporting information:  crystallographic information; 3D view; checkCIF report

## Figures and Tables

**Table 1:** Hydrogen-bond geometry (Å, °)

*D*—H⋯*A*	*D*—H	H⋯*A*	*D*⋯*A*	*D*—H⋯*A*
C3—H3⋯O2^i^	0.93	2.43	3.273 (6)	150
C9—H9⋯O2^ii^	0.93	2.58	3.278 (5)	132
C9—H9⋯O1^ii^	0.93	2.65	3.315 (5)	130

Symmetry codes: (i) 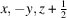; (ii) 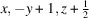.

**Table 2:** Experimental details

Crystal data	
Chemical formula	[Ag_2_(C_21_H_15_N_3_)(CF_3_O_3_S)_2_]
*M*_r_	1132.60
Crystal system, space group	Orthorhombic, *Pbcn*
Temperature (K)	293
*a*, *b*, *c* (Å)	21.3041 (10), 11.7253 (4), 17.3544 (8)
*V* (Å^3^)	4335.1 (3)
*Z*	4
Radiation type	Mo *K*α
μ (mm^−1^)	1.08
Crystal size (mm)	0.35 × 0.1 × 0.06

Data collection	
Diffractometer	Rigaku XtaLAB Mini II
Absorption correction	Multi-scan (*CrysAlis PRO*; Rigaku OD, 2022 ▸)
*T*_min_, *T*_max_	0.700, 1.000
No. of measured, independent and observed [*I* > 2σ(*I*)] reflections	26970, 6431, 3745
*R*_int_	0.038
(sin θ/λ)_max_ (Å^−1^)	0.718

Refinement	
*R*[*F*^2^ > 2σ(*F*^2^)], *wR*(*F*^2^), *S*	0.047, 0.159, 0.92
No. of reflections	6431
No. of parameters	298
H-atom treatment	H-atom parameters constrained
Δρ_max_, Δρ_min_ (e Å^−3^)	0.57, −0.79

Computer programs: *CrysAlis PRO* (Rigaku OD, 2022 ▸), *SHELXT2018* (Sheldrick, 2015*a* ▸), *SHELXL2025* (Sheldrick, 2015*b* ▸) and *OLEX2* (Dolomanov *et al.*, 2009 ▸).

## full crystallographic data

### Bis(µ-6-phenyl-2,2':6',2''-terpyridine)bis[(trifluoromethanesulfonato)silver(I)] Crystal data

**Table:**

[Ag_2_(C_21_H_15_N_3_)(CF_3_O_3_S)_2_]	*D*_x_ = 1.735 Mg m^−^^3^
*M**_r_* = 1132.60	Mo *K*α radiation, λ = 0.71073 Å
Orthorhombic, *P**b**c**n*	Cell parameters from 5625 reflections
*a* = 21.3041 (10) Å	θ = 2.1–24.4°
*b* = 11.7253 (4) Å	µ = 1.08 mm^−^^1^
*c* = 17.3544 (8) Å	*T* = 293 K
*V* = 4335.1 (3) Å^3^	Block, colourless
*Z* = 4	0.35 × 0.1 × 0.06 mm
*F*(000) = 2256	

### Bis(µ-6-phenyl-2,2':6',2''-terpyridine)bis[(trifluoromethanesulfonato)silver(I)] Data collection

**Table:**

Rigaku XtaLAB Mini II diffractometer	6431 independent reflections
Radiation source: fine-focus sealed X-ray tube, Rigaku (Mo) X-ray Source	3745 reflections with *I* > 2σ(*I*)
Graphite monochromator	*R*_int_ = 0.038
Detector resolution: 10.0000 pixels mm^-1^	θ_max_ = 30.7°, θ_min_ = 2.3°
ω scans	*h* = −30→26
Absorption correction: multi-scan (CrysAlis PRO; Rigaku OD, 2022)	*k* = −16→16
*T*_min_ = 0.700, *T*_max_ = 1.000	*l* = −24→19
26970 measured reflections	

### Bis(µ-6-phenyl-2,2':6',2''-terpyridine)bis[(trifluoromethanesulfonato)silver(I)] Refinement

**Table:**

Refinement on *F*^2^	Primary atom site location: dual
Least-squares matrix: full	Hydrogen site location: inferred from neighbouring sites
*R*[*F*^2^ > 2σ(*F*^2^)] = 0.047	H-atom parameters constrained
*wR*(*F*^2^) = 0.159	*w* = 1/[σ^2^(*F*_o_^2^) + (0.1*P*)^2^] where *P* = (*F*_o_^2^ + 2*F*_c_^2^)/3
*S* = 0.92	(Δ/σ)_max_ = 0.001
6431 reflections	Δρ_max_ = 0.57 e Å^−^^3^
298 parameters	Δρ_min_ = −0.79 e Å^−^^3^
0 restraints	

### Bis(µ-6-phenyl-2,2':6',2''-terpyridine)bis[(trifluoromethanesulfonato)silver(I)] Special details

**Table:**

Geometry. All esds (except the esd in the dihedral angle between two l.s. planes) are estimated using the full covariance matrix. The cell esds are taken into account individually in the estimation of esds in distances, angles and torsion angles; correlations between esds in cell parameters are only used when they are defined by crystal symmetry. An approximate (isotropic) treatment of cell esds is used for estimating esds involving l.s. planes.
Refinement. H atoms were placed in calculated positions, with C–H = 0.93 Å, and refined using a riding model, with *U*_iso_(H) = 1.2*U*_eq_(C).

### Bis(µ-6-phenyl-2,2':6',2''-terpyridine)bis[(trifluoromethanesulfonato)silver(I)] Fractional atomic coordinates and isotropic or equivalent isotropic displacement parameters (Å^2^)

**Table:**

	*x*	*y*	*z*	*U*_iso_*/*U*_eq_	
Ag1	0.55847 (2)	0.37117 (2)	0.71012 (2)	0.04187 (12)	
S1	0.63201 (6)	0.16608 (9)	0.55585 (6)	0.0504 (3)	
O2	0.63818 (18)	0.1843 (3)	0.47493 (17)	0.0703 (10)	
N2	0.52874 (14)	0.4296 (2)	0.86920 (16)	0.0349 (7)	
N3	0.43146 (14)	0.5640 (2)	0.81989 (18)	0.0366 (7)	
N1	0.57184 (17)	0.2271 (3)	0.79332 (17)	0.0424 (8)	
O1	0.6101 (2)	0.2597 (3)	0.59889 (19)	0.0825 (12)	
C5	0.57356 (19)	0.2413 (3)	0.8704 (2)	0.0428 (9)	
C15	0.3861 (2)	0.6287 (3)	0.7889 (2)	0.0439 (9)	
C16	0.33765 (19)	0.5713 (3)	0.7428 (3)	0.0447 (9)	
C10	0.52524 (18)	0.5368 (3)	0.8959 (2)	0.0378 (8)	
O3	0.6053 (3)	0.0596 (3)	0.5772 (2)	0.1148 (17)	
C11	0.47793 (19)	0.6128 (3)	0.8607 (2)	0.0390 (8)	
C6	0.57040 (18)	0.3592 (3)	0.9001 (2)	0.0401 (9)	
C4	0.5773 (3)	0.1487 (4)	0.9198 (3)	0.0651 (14)	
H4	0.578236	0.160036	0.972827	0.078*	
C1	0.5747 (2)	0.1201 (3)	0.7656 (3)	0.0531 (11)	
H1	0.573638	0.108782	0.712619	0.064*	
C9	0.56347 (19)	0.5729 (4)	0.9560 (2)	0.0501 (11)	
H9	0.560162	0.647151	0.974331	0.060*	
C14	0.3842 (3)	0.7457 (4)	0.8025 (3)	0.0607 (13)	
H14	0.351214	0.789498	0.783776	0.073*	
C7	0.6099 (2)	0.3925 (4)	0.9606 (2)	0.0524 (11)	
H7	0.638705	0.341642	0.981709	0.063*	
C12	0.4795 (2)	0.7297 (3)	0.8721 (2)	0.0551 (12)	
H12	0.512633	0.763195	0.898656	0.066*	
C17	0.3135 (2)	0.6216 (4)	0.6760 (3)	0.0582 (12)	
H17	0.327801	0.693085	0.660634	0.070*	
C21	0.3146 (2)	0.4648 (4)	0.7633 (3)	0.0523 (10)	
H21	0.328688	0.430119	0.808279	0.063*	
C3	0.5797 (3)	0.0418 (4)	0.8908 (3)	0.0798 (17)	
H3	0.581669	−0.020786	0.923677	0.096*	
C20	0.2711 (2)	0.4099 (5)	0.7176 (3)	0.0697 (14)	
H20	0.256916	0.337755	0.731366	0.084*	
C8	0.6052 (2)	0.5010 (4)	0.9877 (3)	0.0558 (11)	
H8	0.630689	0.525308	1.028020	0.067*	
C18	0.2689 (3)	0.5661 (6)	0.6329 (3)	0.0784 (16)	
H18	0.252173	0.601509	0.589576	0.094*	
C2	0.5792 (3)	0.0269 (4)	0.8136 (3)	0.0674 (14)	
H2	0.581911	−0.046140	0.792846	0.081*	
C19	0.2489 (2)	0.4599 (6)	0.6529 (3)	0.0799 (16)	
H19	0.220032	0.421740	0.622141	0.096*	
C13	0.4315 (3)	0.7954 (4)	0.8438 (3)	0.0652 (14)	
H13	0.431054	0.873627	0.852724	0.078*	
F1	0.7124 (3)	0.1423 (6)	0.6626 (3)	0.207 (3)	
F3	0.7404 (3)	0.0703 (7)	0.5574 (3)	0.214 (3)	
C22	0.7109 (5)	0.1506 (10)	0.5893 (5)	0.129 (3)	
F2	0.7406 (3)	0.2552 (8)	0.5666 (4)	0.250 (4)	

### Bis(µ-6-phenyl-2,2':6',2''-terpyridine)bis[(trifluoromethanesulfonato)silver(I)] Atomic displacement parameters (Å^2^)

**Table:**

	*U*^11^	*U*^22^	*U*^33^	*U*^12^	*U*^13^	*U*^23^
Ag1	0.0483 (2)	0.03713 (17)	0.04016 (18)	0.00238 (13)	−0.00214 (13)	0.00872 (11)
S1	0.0657 (7)	0.0467 (5)	0.0389 (6)	0.0138 (5)	−0.0019 (5)	−0.0025 (4)
O2	0.115 (3)	0.0550 (18)	0.0406 (17)	0.019 (2)	0.0045 (18)	−0.0009 (14)
N2	0.0374 (17)	0.0374 (15)	0.0298 (15)	−0.0032 (14)	−0.0029 (13)	−0.0024 (12)
N3	0.0409 (19)	0.0332 (15)	0.0356 (16)	0.0001 (13)	0.0039 (14)	−0.0032 (12)
N1	0.053 (2)	0.0381 (16)	0.0358 (17)	0.0046 (15)	0.0000 (14)	0.0043 (13)
O1	0.131 (4)	0.062 (2)	0.054 (2)	0.034 (2)	0.011 (2)	−0.0065 (16)
C5	0.041 (2)	0.047 (2)	0.040 (2)	0.0065 (17)	−0.0031 (17)	0.0083 (17)
C15	0.048 (2)	0.0402 (19)	0.044 (2)	0.0071 (18)	0.0108 (18)	0.0025 (16)
C16	0.036 (2)	0.049 (2)	0.049 (2)	0.0111 (18)	0.0039 (18)	−0.0010 (19)
C10	0.040 (2)	0.0407 (19)	0.0327 (18)	−0.0107 (17)	0.0041 (16)	−0.0069 (15)
O3	0.186 (5)	0.077 (3)	0.081 (3)	−0.052 (3)	0.022 (3)	0.002 (2)
C11	0.046 (2)	0.0348 (18)	0.036 (2)	−0.0051 (16)	0.0078 (16)	−0.0026 (14)
C6	0.039 (2)	0.050 (2)	0.0320 (19)	−0.0040 (17)	−0.0026 (15)	0.0033 (15)
C4	0.086 (4)	0.062 (3)	0.047 (3)	0.015 (3)	−0.008 (2)	0.017 (2)
C1	0.070 (3)	0.040 (2)	0.050 (2)	0.010 (2)	0.002 (2)	0.0018 (18)
C9	0.049 (3)	0.056 (2)	0.046 (2)	−0.017 (2)	−0.0024 (19)	−0.0128 (19)
C14	0.066 (3)	0.040 (2)	0.077 (3)	0.011 (2)	0.003 (3)	−0.002 (2)
C7	0.046 (2)	0.071 (3)	0.041 (2)	−0.001 (2)	−0.0093 (18)	0.001 (2)
C12	0.070 (3)	0.035 (2)	0.060 (3)	−0.014 (2)	−0.003 (2)	−0.0083 (18)
C17	0.058 (3)	0.060 (3)	0.057 (3)	0.016 (2)	0.002 (2)	0.006 (2)
C21	0.039 (2)	0.057 (3)	0.061 (3)	0.000 (2)	0.002 (2)	0.004 (2)
C3	0.117 (5)	0.053 (3)	0.070 (4)	0.019 (3)	−0.009 (3)	0.027 (3)
C20	0.048 (3)	0.075 (3)	0.086 (4)	−0.010 (3)	0.000 (3)	−0.009 (3)
C8	0.048 (3)	0.079 (3)	0.041 (2)	−0.018 (2)	−0.010 (2)	−0.006 (2)
C18	0.061 (4)	0.113 (5)	0.061 (3)	0.023 (4)	−0.013 (3)	−0.001 (3)
C2	0.086 (4)	0.038 (2)	0.078 (3)	0.014 (2)	0.005 (3)	0.005 (2)
C19	0.044 (3)	0.123 (5)	0.073 (4)	−0.001 (3)	−0.015 (3)	−0.025 (4)
C13	0.090 (4)	0.0279 (19)	0.078 (3)	0.002 (2)	0.003 (3)	−0.006 (2)
F1	0.163 (5)	0.365 (9)	0.092 (4)	0.131 (5)	−0.056 (3)	−0.014 (4)
F3	0.167 (5)	0.332 (8)	0.144 (5)	0.182 (6)	0.015 (4)	−0.002 (5)
C22	0.102 (7)	0.201 (10)	0.084 (6)	0.045 (7)	−0.001 (5)	0.003 (6)
F2	0.129 (5)	0.393 (12)	0.228 (7)	−0.107 (7)	−0.015 (5)	0.004 (7)

### Bis(µ-6-phenyl-2,2':6',2''-terpyridine)bis[(trifluoromethanesulfonato)silver(I)] Geometric parameters (Å, º)

**Table:**

Ag1—Ag1^i^	2.8501 (8)	C1—H1	0.9300
Ag1—N2^i^	2.412 (3)	C1—C2	1.377 (6)
Ag1—N3^i^	2.330 (3)	C9—H9	0.9300
Ag1—N1	2.240 (3)	C9—C8	1.343 (6)
Ag1—O1	2.578 (3)	C14—H14	0.9300
S1—O2	1.427 (3)	C14—C13	1.366 (7)
S1—O1	1.407 (3)	C7—H7	0.9300
S1—O3	1.421 (4)	C7—C8	1.361 (6)
S1—C22	1.786 (9)	C12—H12	0.9300
N2—C10	1.342 (4)	C12—C13	1.372 (6)
N2—C6	1.326 (5)	C17—H17	0.9300
N3—C15	1.342 (5)	C17—C18	1.373 (7)
N3—C11	1.345 (5)	C21—H21	0.9300
N1—C5	1.349 (5)	C21—C20	1.380 (7)
N1—C1	1.345 (5)	C3—H3	0.9300
C5—C6	1.476 (5)	C3—C2	1.353 (7)
C5—C4	1.386 (5)	C20—H20	0.9300
C15—C16	1.468 (6)	C20—C19	1.351 (7)
C15—C14	1.393 (5)	C8—H8	0.9300
C16—C17	1.399 (6)	C18—H18	0.9300
C16—C21	1.388 (6)	C18—C19	1.362 (8)
C10—C11	1.478 (5)	C2—H2	0.9300
C10—C9	1.389 (5)	C19—H19	0.9300
C11—C12	1.385 (5)	C13—H13	0.9300
C6—C7	1.402 (5)	F1—C22	1.277 (9)
C4—H4	0.9300	F3—C22	1.260 (9)
C4—C3	1.351 (7)	C22—F2	1.436 (11)
			
N2^i^—Ag1—Ag1^i^	66.66 (7)	N1—C1—C2	121.9 (5)
N2^i^—Ag1—O1	92.60 (11)	C2—C1—H1	119.1
N3^i^—Ag1—Ag1^i^	100.90 (8)	C10—C9—H9	119.8
N3^i^—Ag1—N2^i^	70.59 (10)	C8—C9—C10	120.4 (4)
N3^i^—Ag1—O1	106.58 (11)	C8—C9—H9	119.8
N1—Ag1—Ag1^i^	78.35 (9)	C15—C14—H14	120.3
N1—Ag1—N2^i^	132.85 (12)	C13—C14—C15	119.3 (5)
N1—Ag1—N3^i^	149.76 (12)	C13—C14—H14	120.3
N1—Ag1—O1	92.64 (11)	C6—C7—H7	120.8
O1—Ag1—Ag1^i^	137.45 (10)	C8—C7—C6	118.4 (4)
O2—S1—C22	104.4 (3)	C8—C7—H7	120.8
O1—S1—O2	115.9 (2)	C11—C12—H12	120.4
O1—S1—O3	114.4 (3)	C13—C12—C11	119.1 (4)
O1—S1—C22	102.7 (4)	C13—C12—H12	120.4
O3—S1—O2	115.2 (2)	C16—C17—H17	119.8
O3—S1—C22	101.7 (4)	C18—C17—C16	120.3 (5)
C10—N2—Ag1^i^	114.9 (2)	C18—C17—H17	119.8
C6—N2—Ag1^i^	124.7 (2)	C16—C21—H21	119.7
C6—N2—C10	118.7 (3)	C20—C21—C16	120.6 (5)
C15—N3—Ag1^i^	121.7 (3)	C20—C21—H21	119.7
C15—N3—C11	120.1 (3)	C4—C3—H3	120.4
C11—N3—Ag1^i^	117.6 (2)	C4—C3—C2	119.3 (4)
C5—N1—Ag1	123.3 (3)	C2—C3—H3	120.4
C1—N1—Ag1	118.6 (3)	C21—C20—H20	119.7
C1—N1—C5	117.9 (4)	C19—C20—C21	120.7 (5)
S1—O1—Ag1	159.1 (2)	C19—C20—H20	119.7
N1—C5—C6	117.4 (3)	C9—C8—C7	119.6 (4)
N1—C5—C4	121.2 (4)	C9—C8—H8	120.2
C4—C5—C6	121.4 (4)	C7—C8—H8	120.2
N3—C15—C16	117.8 (3)	C17—C18—H18	119.6
N3—C15—C14	120.5 (4)	C19—C18—C17	120.7 (5)
C14—C15—C16	121.6 (4)	C19—C18—H18	119.6
C17—C16—C15	121.1 (4)	C1—C2—H2	120.1
C21—C16—C15	121.4 (4)	C3—C2—C1	119.8 (5)
C21—C16—C17	117.6 (4)	C3—C2—H2	120.1
N2—C10—C11	117.4 (3)	C20—C19—C18	120.0 (5)
N2—C10—C9	120.8 (4)	C20—C19—H19	120.0
C9—C10—C11	121.8 (4)	C18—C19—H19	120.0
N3—C11—C10	117.6 (3)	C14—C13—C12	119.8 (4)
N3—C11—C12	120.9 (4)	C14—C13—H13	120.1
C12—C11—C10	121.4 (4)	C12—C13—H13	120.1
N2—C6—C5	118.2 (3)	F1—C22—S1	110.9 (6)
N2—C6—C7	122.1 (4)	F1—C22—F2	109.0 (10)
C7—C6—C5	119.6 (4)	F3—C22—S1	113.8 (8)
C5—C4—H4	120.0	F3—C22—F1	111.6 (8)
C3—C4—C5	120.0 (5)	F3—C22—F2	107.2 (9)
C3—C4—H4	120.0	F2—C22—S1	103.9 (6)
N1—C1—H1	119.1		
			
Ag1^i^—N2—C10—C11	−13.4 (4)	C15—C16—C21—C20	−177.2 (4)
Ag1^i^—N2—C10—C9	164.6 (3)	C15—C14—C13—C12	−0.9 (8)
Ag1^i^—N2—C6—C5	15.1 (5)	C16—C15—C14—C13	−178.0 (4)
Ag1^i^—N2—C6—C7	−163.3 (3)	C16—C17—C18—C19	−2.2 (8)
Ag1^i^—N3—C15—C16	7.7 (5)	C16—C21—C20—C19	−1.6 (7)
Ag1^i^—N3—C15—C14	−174.5 (3)	C10—N2—C6—C5	179.7 (3)
Ag1^i^—N3—C11—C10	−11.5 (4)	C10—N2—C6—C7	1.3 (6)
Ag1^i^—N3—C11—C12	171.5 (3)	C10—C11—C12—C13	−174.3 (4)
Ag1—N1—C5—C6	−4.0 (5)	C10—C9—C8—C7	0.1 (7)
Ag1—N1—C5—C4	175.2 (3)	O3—S1—O1—Ag1	18.4 (9)
Ag1—N1—C1—C2	−176.2 (4)	O3—S1—C22—F1	−64.9 (9)
O2—S1—O1—Ag1	156.1 (7)	O3—S1—C22—F3	61.9 (9)
O2—S1—C22—F1	175.0 (7)	O3—S1—C22—F2	178.1 (6)
O2—S1—C22—F3	−58.2 (9)	C11—N3—C15—C16	178.0 (3)
O2—S1—C22—F2	58.0 (7)	C11—N3—C15—C14	−4.1 (6)
N2—C10—C11—N3	17.0 (5)	C11—C10—C9—C8	178.7 (4)
N2—C10—C11—C12	−166.0 (4)	C11—C12—C13—C14	−2.4 (7)
N2—C10—C9—C8	0.8 (6)	C6—N2—C10—C11	−179.5 (3)
N2—C6—C7—C8	−0.4 (6)	C6—N2—C10—C9	−1.4 (5)
N3—C15—C16—C17	−141.8 (4)	C6—C5—C4—C3	179.5 (5)
N3—C15—C16—C21	37.5 (6)	C6—C7—C8—C9	−0.3 (7)
N3—C15—C14—C13	4.2 (7)	C4—C5—C6—N2	−134.9 (4)
N3—C11—C12—C13	2.6 (6)	C4—C5—C6—C7	43.6 (6)
N1—C5—C6—N2	44.3 (5)	C4—C3—C2—C1	−1.6 (9)
N1—C5—C6—C7	−137.2 (4)	C1—N1—C5—C6	180.0 (4)
N1—C5—C4—C3	0.4 (8)	C1—N1—C5—C4	−0.8 (7)
N1—C1—C2—C3	1.2 (9)	C9—C10—C11—N3	−161.0 (3)
O1—S1—C22—F1	53.7 (9)	C9—C10—C11—C12	16.0 (6)
O1—S1—C22—F3	−179.5 (8)	C14—C15—C16—C17	40.4 (6)
O1—S1—C22—F2	−63.2 (6)	C14—C15—C16—C21	−140.3 (4)
C5—N1—C1—C2	0.1 (7)	C17—C16—C21—C20	2.1 (6)
C5—C6—C7—C8	−178.8 (4)	C17—C18—C19—C20	2.8 (9)
C5—C4—C3—C2	0.9 (9)	C21—C16—C17—C18	−0.2 (7)
C15—N3—C11—C10	177.7 (3)	C21—C20—C19—C18	−0.9 (8)
C15—N3—C11—C12	0.7 (6)	C22—S1—O1—Ag1	−90.8 (8)
C15—C16—C17—C18	179.1 (4)		

Symmetry code: (i) −*x*+1, *y*, −*z*+3/2.

### Bis(µ-6-phenyl-2,2':6',2''-terpyridine)bis[(trifluoromethanesulfonato)silver(I)] Hydrogen-bond geometry (Å, º)

**Table:**

*D*—H···*A*	*D*—H	H···*A*	*D*···*A*	*D*—H···*A*
C3—H3···O2^ii^	0.93	2.43	3.273 (6)	150
C9—H9···O2^iii^	0.93	2.58	3.278 (5)	132
C9—H9···O1^iii^	0.93	2.65	3.315 (5)	130

Symmetry codes: (ii) *x*, −*y*, *z*+1/2; (iii) *x*, −*y*+1, *z*+1/2.
